# Dilated Cardiomyopathy Induced by Chronic Starvation and Selenium Deficiency

**DOI:** 10.1155/2016/8305895

**Published:** 2016-11-22

**Authors:** Soham Dasgupta, Ashraf M. Aly

**Affiliations:** ^1^Department of Pediatrics, University of Texas Medical Branch, Galveston, TX 77555, USA; ^2^Department of Pediatric Cardiology, University of Texas Medical Branch, Galveston, TX 77555, USA

## Abstract

Protein energy malnutrition (PEM) has been rarely documented as a cause of cardiovascular abnormalities, including dilated cardiomyopathy. Selenium is responsible for antioxidant defense mechanisms in cardiomyocytes, and its deficiency in the setting of PEM and disease related malnutrition (DRM) may lead to exacerbation of the dilated cardiomyopathy. We report a rare case of a fourteen-year-old boy who presented with symptoms of congestive heart failure due to DRM and PEM (secondary to chronic starvation) along with severe selenium deficiency. An initial echocardiogram showed severely depressed systolic function consistent with dilated cardiomyopathy. Aggressive nutritional support and replacement of selenium and congestive heart failure medications that included diuretics and ACE inhibitors with the addition of carvedilol led to normalization of the cardiac function within four weeks. He continues to have significant weight gain and is currently completely asymptomatic from a cardiovascular standpoint.

## 1. Introduction

The World Health Organization (WHO) defines malnutrition as “the cellular imbalance between the supply of nutrients and energy and the body's demand for them to ensure growth, maintenance, and specific functions [1].” The term protein energy malnutrition (PEM) applies to a group of related disorders that include marasmus, kwashiorkor, and intermediate states of marasmus-kwashiorkor. Marasmus involves inadequate intake of protein and calories and is characterized by emaciation while kwashiorkor involves normal caloric intake but inadequate protein intake. We report a rare case of dilated cardiomyopathy caused by severe malnutrition combined with selenium deficiency in a teenager. The malnutrition seen in this patient can be more accurately defined as “disease or injury related malnutrition” and is commonly seen in the setting of major infection, burns, or trauma [2].

## 2. Case Report

A fourteen-year-old boy from South America was transferred to the burn unit at our institution for severe malnutrition. The patient had sustained second-degree burns (>25% BSA) two years prior to admission. He did not receive adequate nutrition and medical care because of poverty and limited resources in his home country. He was noted to be severely cachectic and malnourished at a local hospital and was transferred to the United States by a charity organization for higher level of care.

Initial assessment was significant for a weight of 19 kg (<3rd percentile on WHO growth chart), height of 141 cm (BMI 9.6 kg/m^2^, *z* score −12.42), blood pressure of 92/63 mmHg, and a heart rate of 140 bpm. He experienced shortness of breath with minimal physical activity. Physical examination revealed severe cachexia with atrophy of all muscles without peripheral edema. He was also noted to be moderately dehydrated. Initial investigations were significant for mild hyponatremia [133 mmol/L (NL 135–145 mmol/L)], severe hypoalbuminemia [0.2 g/dL (NL 3.5–5.0 g/dL)], hypocalcemia [ionized calcium 3.2 mg/dL (NL 4.5–5.3 mg/dL)], severe iron deficiency anemia [hemoglobin/hematocrit 6.7 gm/dL/24.8%, MCV 69.3 FL], and selenium deficiency [32 ug/L]. The reported normal serum selenium levels in the United States ranges from 50 to 150 ug/L [3] while the normal serum levels of selenium in his country of origin is reported to be 80.6–199 ug/L [4]. The carnitine level was normal.

**Figure 1:**
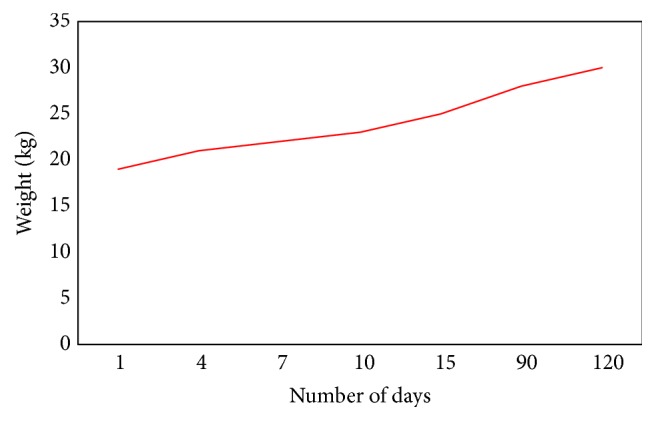
A diagram showing the increase in body weight with time.

Immediate intravenous fluid resuscitation was initiated. Since he was severely malnourished and had limited ability for oral intake, high calorie enteral nutrition was administered via a nasogastric tube. An initial echocardiogram (echo) showed a globular dilated left ventricle with a severely depressed systolic function [ejection fraction (EF) < 25%] consistent with dilated cardiomyopathy. He was initially treated with furosemide and enalapril, and carvedilol was added a week later since there was minimal improvement in cardiac function in addition to selenium replacement [200 mcg (2.5 umol) twice daily intravenously]. He received two blood transfusions and repeated albumin infusions within the first five days. One week later, his cardiac function dramatically improved [EF 46%]. His selenium level, as well as other labs, normalized within two weeks. The cardiac function normalized within four weeks. His weight continued to increase and reached 30 kg [BMI 15.1 kg/m^2^, *z* score −2.40] within four months (Figure 1). All cardiac medications were then discontinued. The patient's stamina continues to improve, and he is able to tolerate full oral intake.

## 3. Discussion

Malnutrition is a significant cause of morbidity and mortality in developing countries. It is the direct cause of over 300,000 deaths annually and is indirectly responsible for about half of all deaths in children [5]. The median prevalence of underweight children worldwide in the year 2012 was 15% with 3% of them having severe muscle wasting [6]. Malnutrition may be secondary to a variety of reasons including poverty, pathologic states, political situations in certain countries, and even acts of rebellion in order to achieve certain goals. Specifically, “disease related malnutrition” is defined as malnutrition in the setting of severe inflammation and is commonly seen in the setting of major infection, burns, or trauma. Critical illness or injury promotes an acute inflammatory response that has a rapid catabolic effect on lean body mass [7]. The inflammatory condition in most diseases is chronic in nature and loss of muscle mass and function may occur over months to years. This form of malnutrition is partially attributable to a decrease in nutrient intake and to the effect of the inflammatory state on metabolism. The malnutrition seen in this case was both disease related and secondary to starvation as a result of his disease and poverty.

Children suffering from severe malnutrition may exhibit cardiovascular abnormalities including hypotension, cardiac arrhythmias, cardiomyopathy, cardiac failure, and even sudden death [8]. The cardiac myocytes atrophy during starvation similar to other muscles in the body [8]. Kerpel-Fronius and Varga showed a 60% decrease in the weight of the heart in an autopsy study [9]. Ultrastructural features of rats with PEM showed hyalinization and vacuolization of cardiac muscle fibers, loss of cross striations and myofibrils, small foci of necrosis, interstitial fibrosis, and mononuclear cell infiltration [10]. Histological studies of human myocardium in patients with PEM show atrophy of the muscle fibers with interstitial edema. The heart has also been reported to be thin-walled, pale, and flabby on gross examination in other autopsy studies of children with malnutrition [11].

Echocardiographic studies have shown decreased left ventricular (LV) mass in patients with PEM. Singh et al. reported that LV systolic functions were reduced especially in children with a loss in bodyweight of more than 40% of expected weight [12]. A recent study showed that parameters of LV systolic function were significantly reduced in patients with PEM as compared to controls [13]. PEM has been established as an independent risk factor for mortality in patients with heart failure [14].

**Figure 2:**
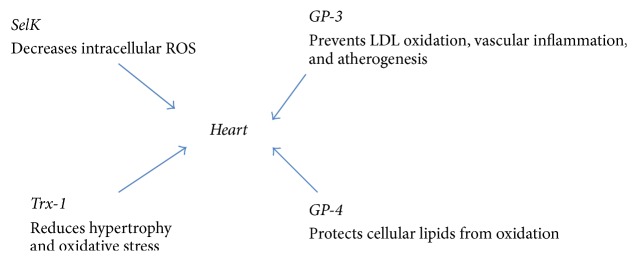
A diagram showing the actions of various selenoproteins on the cardiac myocytes. SelK: selenoprotein K. GP-3: glutathione peroxidase-3. Trx-1: thioredoxin reductase-1. GP-4: glutathione peroxidase-4. ROS: reactive oxygen species. LDL: low density lipoprotein.

Chinese investigators first showed that selenium deficiency is one of the principal factors responsible for Keshan disease, a dilated cardiomyopathy that affects people living in rural areas where selenium is deficient [15]. Selenium mediates its effects via incorporation into selenoproteins (Figure 2). Selenium-dependent enzymes mediate a wide range of biological functions such as regulation of the inflammatory response and proliferation/differentiation of several immune cells.

Selenium is also important in the body's antioxidant defense mechanism [16]. When incorporated into the various selenoenzymes, selenium increases antioxidant capacity and suppresses the production of interleukins and tumor necrosis factor alpha (TNF-*α*). Lu et al. provided the first evidence that selenoproteins contribute to the antioxidant defense mechanisms in cardiomyocytes. Venardos and colleagues demonstrated that selenium deficiency leads to myocardial injury secondary to increased protein and lipid peroxidation in a rat model. These data were supported by the experiments of Tanguy et al., who showed that selenium deficiency in rats lead to myocardial damage and altered recovery of cardiac function.

Given these findings, various experimental studies have aimed to limit myocardial injury through selenium supplementation. The Venardos group showed significantly more myocardial injury in rats fed with a low selenium diet. Furthermore, Tanguy and colleagues confirmed selenium's protective characteristics by demonstrating (a) an improved cardiac function recovery, (b) a significantly reduced infarct size, and (c) a decreased incidence of postischemic ventricular arrhythmias in rats that received the highest selenium intake.

The patient we described had some features consistent with marasmus (Table 1). He also had evidence of dilated cardiomyopathy based on congestive heart failure symptoms and echocardiographic findings. He responded well to standard therapy for dilated cardiomyopathy with furosemide (preload reduction), enalapril (afterload reduction), and carvedilol (antioxidant effects). Carvedilol is a nonselective beta blocker which suppresses reactive oxygen species and has antioxidant and anti-inflammatory effects [17]. Selenium supplementation was added for its documented deficiency in addition to providing adequate nutritional support. There was a remarkable weight gain from 19 kg to 25 kg in just 2 weeks and he continues to gain more weight. He is currently asymptomatic from a cardiovascular standpoint and is tolerating complete oral intake.

## 4. Conclusion

Cardiac injury leading to dilated cardiomyopathy in cases of severe malnutrition is rare but documented in the literature. During the treatment of patients with malnutrition, levels of trace elements (specially selenium) should be checked. Adequate nutrition and replacement of deficient elements should be initiated as soon as possible. Congestive heart failure and dilated cardiomyopathy in these patients seem to respond well to preload and afterload reduction with the addition of carvedilol as an antioxidant.

## Figures and Tables

**Table 1:** A comparison between marasmus and kwashiorkor.

Marasmus (PEM)	Kwashiorkor
Severe deficiency of all nutrients and inadequate caloric intake	Severe protein deficiency but normal caloric intake
Peripheral edema is absent	Peripheral edema is present
Hair changes absent	Hair changes common (sparse and easily pulled out)
Skin is dry and wrinkled but no dermatosis	Dermatosis, flaky paint appearance of skin
Voracious appetite	Poor appetite
Absent subcutaneous fat	Reduced subcutaneous fat
Fatty liver uncommon	Fatty liver common
Better prognosis	Worse prognosis

## Abbreviations

WHO: World Health Organization
PEM: Protein energy malnutrition
BSA: Body surface area
BMI: Body mass index
LV: Left ventricular
TNF-*α*: Tumor necrosis factor-*α*.
